# Supplementary material for: Health Assessment of Electronic Waste Workers in Chile: Participant Characterization
Source: Int J Environ Res Public Health. 2019 Jan 29;16(3):386. doi: 10.3390/ijerph16030386 (PMC6388190; doi:10.3390/ijerph16030386)
Supplement: Supplementary file 1 [file ijerph-16-00386-s001.pdf]

Table S 1. Symptoms experienced in the last 2 weeks of the study population, by job type and site

	**Informal**		**Formal**	
	**Santiago N = 53**	**Temuco N = 25**	**Recycling company N = 15**	**p-value***
- *Skin rashes (%)*				
Rarely or never	85	80	87	0.842
Occasionally	9.4	12	13	
Always or frequently	5.7	8	0	
- *Headache or dizziness (%)*				
Rarely or never	47	52	67	0.674
Occasionally	38	44	27	
Always or frequently	13	4	6.7	
- *Shaking or tremors (%)*				
Rarely or never	93	92	100	0.741
Occasionally	5.7	8	0	
Always or frequently	1.9	0	0	
- *Blood in urine (%)*				
Rarely or never	94	100	100	
Occasionally	3.8	0	0	
Always or frequently	1.9	0	0	0.674
- *Blood in stool (%)*				
Rarely or never	98	96	100	0.479
Occasionally	0	4	0	
Always or frequently	1.9	0	0	
- *Breathing problems (%)*				
Rarely or never	68	84	87	0.288
Occasionally	28	12	6.7	
Always or frequently	3.8	4	6.7	
- *Heart beating abnormally (%)*				
Rarely or never	55	88	93	**0.008**
Occasionally	40	12	6.7	
Always or frequently	5.7	0	0	
- *Loose or watery stools (%)*				
Rarely or never	83	88	87	0.408
Occasionally	15	4	6.7	
Always or frequently	1.9	8	6.7	
- *Fever (%)*				
Rarely or never	89	88	100	0.625
Occasionally	9.4	12	0	
Always or frequently	1.9	0	0	
- *Nausea or stomach ache (%)*				
Rarely or never	76	76	80	0.962
Occasionally	21	20	13	
Always or frequently	3.8	4	6.7	

*chi2 for the comparison between formal and informal and categories in the variable

Table S 2. Self-report of stress in the study population, by job type and site

Group	Informal: Santiago N = 53	Informal: Temuco N = 25	Formal: Recycling company N = 15	p-value*
***Stressors by* Cohen's perceived stress scale**				
In the last month, how often have you felt:				
- Unable to control important things in life (%)				
Never	30	40	60	0.388
Almost never	23	20	13	
Sometimes	32	24	27	
Fairly often	13	8	0	
Very often	1.9	8	0	
- Confident about ability to handle problems (%)				
Never	5.7	0	0	0.284
Almost never	5.7	8	0	
Sometimes	7.5	4	13	
Fairly often	51	32	33	
Very often	30	56	53	
- Things were going your way (%)				
Never	3.8	0	6.7	0.301
Almost never	7.5	4	0	
Sometimes	40	16	40	
Fairly often	42	60	40	
Very often	7.5	20	13	
- Couldn't overcome difficulties (%)				
Never	28	40	27	0.619
Almost never	25	32	33	
Sometimes	32	24	40	
Fairly often	11	4	0	
Very often	3.8	0	0	
Other stressors				
- Someone else decide work methods/pace/order (%)				
Never/Almost never	83	68	73	0.359
Occasionally	11	16	6.7	
Always or frequently	5.7	16	20	
- Experience violence or harassment at work (%)				
Never/Almost never	85	72	93	0.066
Occasionally	9.4	24	0	
Always or frequently	5.7	4	0	
Prefer not to answer	0	0	6.7	
- Work interfere with family responsibilities/leisure time (%)				
Never/Almost never	60	60	73	0.88
Occasionally	26	24	20	
Always or frequently	13	16	6.7	
- Income not sufficient to support family (%)				
Never/Almost never	30	64	40	**0.029**
Occasionally	34	16	47	
Always or frequently	36	20	13	

*chi2 for the comparison between formal and informal and categories in the variable
